# Assessing Disease and Mortality among Small Cetaceans Stranded at a World Heritage Site in Southern Brazil

**DOI:** 10.1371/journal.pone.0149295

**Published:** 2016-02-12

**Authors:** Isabela G. Domiciano, Camila Domit, Matt K. Broadhurst, Mariana S. Koch, Ana Paula F. R. L. Bracarense

**Affiliations:** 1 Laboratory of Animal Pathology, Department of Veterinary Preventive Medicine, Universidade Estadual de Londrina, Londrina, Paraná, Brazil; 2 Laboratory of Ecology and Conservation, Centro de Estudos do Mar, Universidade Federal do Paraná, Pontal do Paraná, Paraná, Brazil; 3 NSW Department of Primary Industries, Fisheries Conservation Technology Unit, Coffs Harbour, Australia; 4 Marine and Estuarine Ecology Unit, School of Biological Sciences, University of Queensland, Brisbane, Australia; University of Western Ontario, CANADA

## Abstract

Cetaceans are considered environmental sentinels and their health often reflects either anthropogenic or natural spatio-temporal disturbances. This study investigated the pathological findings and mortality of small cetaceans with the aim of detecting hazards and monitoring health trends in a high-biodiversity area. Between 2007 and 2012, 218 stranded cetaceans were recorded on the Paraná coast, southern Brazil. Fifty-seven (26.1%) of these animals, including 50 *Sotalia guianensis*, 2 *Pontoporia blainvillei*, 2 *Stenella frontalis*, 1 *Stenella longirostris*, 1 *Tursiops truncatus* and 1 *Globicephala melas* were necropsied and samples were collected for histopathology. Causes of death were determined in 46 of the 57 (80.7%) animals and most (30 or 65.2%) were ascribed to anthropogenic activities, including fisheries bycatch (28/30) and trauma (2/30). The remaining 16 fatalities were considered natural, and attributed to pneumonia (10/16), emaciation (3/16), septicemia (1/16), neonatal pathology (1/16) and choking via food obstruction (1/16). Irrespective of the cause, bronchointerstitial pneumonia, associated with parasitism, lymphadenitis and membranous glomerulonephritis were common findings among all fatalities. These results suggest, that while anthropogenic activities are a leading cause of cetacean strandings in Paraná, underlying pre-existing diseases may contribute towards deaths. Although the studied area is considered a biosphere reserve by UNESCO, complex anthropogenic and natural interactions might be occurring, increasing cetacean susceptibility to hazards. This study may help facilitate developing an effective conservation plan for coastal cetaceans focusing on reducing fisheries interactions, habitat degradation and pollution as mechanisms for ultimately increasing species resilience.

## Introduction

Globally, numerous populations of marine mammals have declined in recent decades, largely due to anthropogenic activities [1]. At least a quarter of the world’s cetacean species are classified as endangered, although the situation may be worse because the status of many others species remains unclear [1]. Marine mammals are very sensitive to marine-ecosystem health and they can reflect the magnitude and rapidity of environmental changes [2]. Cetaceans that inhabit coastal zones are particularly vulnerable to either direct or indirect anthropogenic disturbances [3] including, but not limited to: (i) bycatch or predation associated with fishing operations [4,5]; (ii) acoustic pollution (iii) vessel collisions [6,7]; (iv) harmful algal blooms [2]; and (v) the discharge of pathological agents and persistent chemical contaminants from domestic, industrial and agricultural waste [8]. Consequently, health assessments of coastal cetaceans can be used to indirectly monitor the quality of marine ecosystems, investigate the severity of anthropogenic impacts, and identify risks to humans utilizing the same habitats for exploiting natural resources or for recreation [2].

Emerging diseases among wildlife in marine environments can alter population abundances and cause major regime changes within communities [9]. In the last 25 years, at least 10 morbillivirus epidemics, with high associated mortalities, have affected free-ranging pinniped and cetaceans worldwide [10,11]. Diagnosing such infectious diseases and characterizing etiological changes is challenging in free-ranging cetaceans [12]. Pathological examinations of stranded sick or dying marine mammals (often discarded from fishing activities) provide an opportunity to collect data for biological and health assessments [13,14].

Such work is still incipient in many areas, including South America [15]. Nevertheless, several important emerging and recurring conditions affecting different cetacean species, including morbillivirus, fungi and various species of bacteria and parasites have been identified along the Brazilian coast [16,17]. These chronic infectious and parasitic diseases, combined with human impacts, may directly affect the immune and endocrine systems of cetaceans, with consequences ranging from compromised reproductive potential to mortality [18,19].

Over the years, numerous cetaceans, including the Guiana dolphin (*Sotalia guianensis*), bottlenose dolphin (*Tursiops truncates*), franciscana (*Pontoporia blainvillei*) and Atlantic spotted dolphin (*Stenella frontalis*) have been observed feeding and nurturing their calves along the coastal and estuarine areas of Paraná state, Southern Brazil. At the northern limit of this coast is the Paranaguá estuarine complex (PEC); an area comprising part of the last preserved Atlantic rainforest area in South America and with abundant rare and endangered fauna and flora. The region is classified as a biosphere reserve and a World Heritage site by the United Nations Educational, Scientific and Cultural Organization (UNESCO).

Notwithstanding its conservation status, the PEC has been severely impacted by anthropogenic factors, with the port area considered the most important handling site for grain and fertilizers in South America [20]. The region is subjected to continuous and persistent oil spills and trace-element contamination during traffic and unloading of organic matter, as well as shipwrecks [21, 22]. Further, inorganic nutrient levels in untreated waste water discharged from Paranaguá city by far exceed legislated national threshold levels [20], thereby contributing towards the chemical contamination of the PEC.

Given the ecological conditions of the PEC and the local abundance of stranded marine mammals, the main objective of this study was to investigate the pathology and causes of death among small stranded odontocetes. In addition, the study aimed to provide baseline data for the health assessment of coastal dolphin populations, which ultimately might facilitate the future monitoring of spatio-temporal trends throughout the South Atlantic.

## Materials and Methods

### Study area and animals

**Fig 1:**
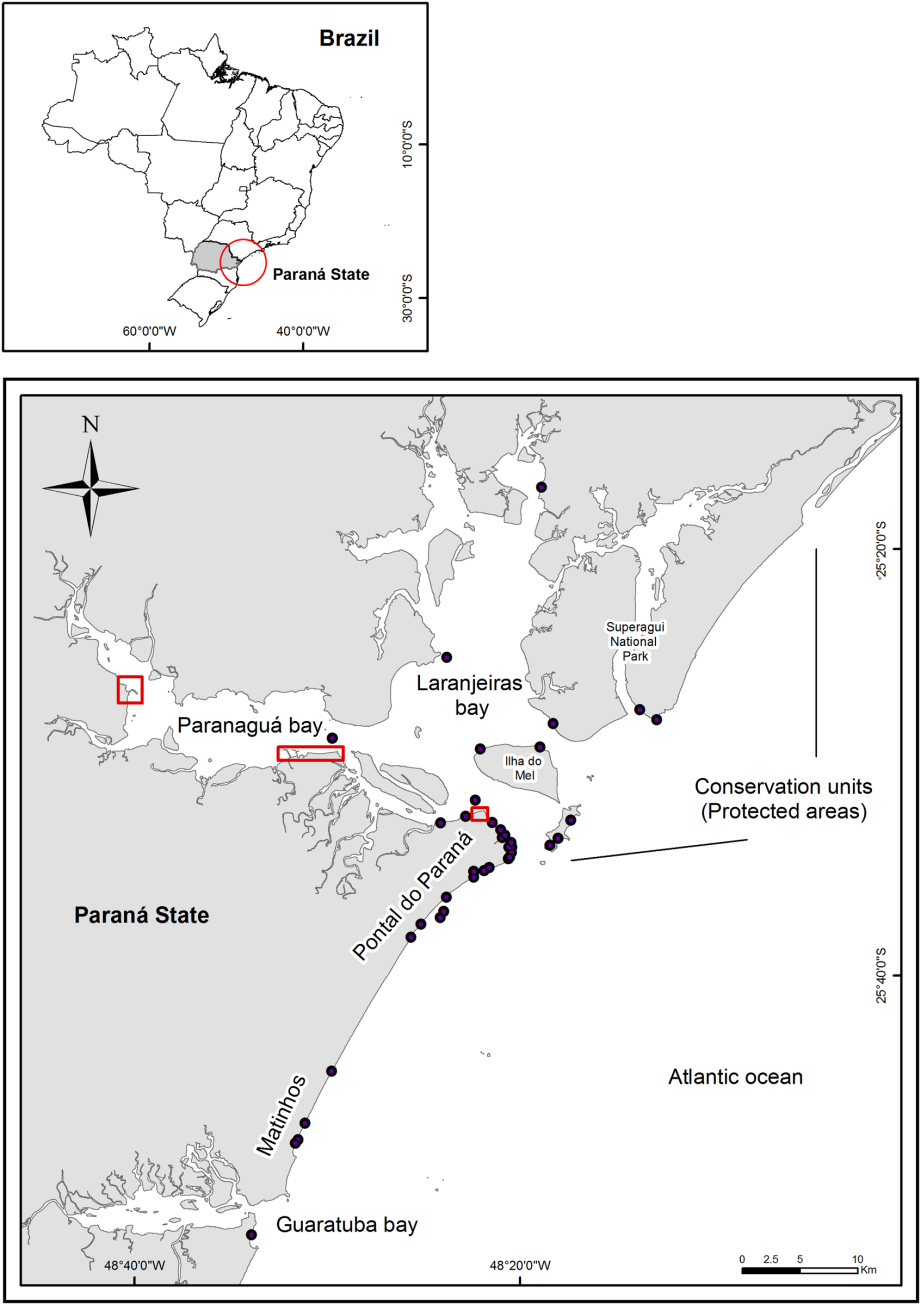
Paranaguá estuarine complex and adjacent coastal area from Pontal do Sul to Matinhos, Paraná, Brazil. Areas with stranded animals are designated by black dots, while harbor zones are within red lines.

The studied area included the PEC and the adjacent 100 km of coastline (25°44’S and 48°29’W). The censused area within the PEC included five major bays (Paranaguá, Antonina, Laranjeiras, Guaraqueçaba, and Pinheiros) and three islands (Mel, Superagui, and Peças) encompassing a total area of 618 km^2^ (Fig 1).

Between July 2007 and November 2012, the ocean beaches and PEC were monitored weekly and monthly, respectively, for any stranded cetaceans. Carcasses were sexed, measured for total length (TL) and classified as fresh, moderate or advanced decomposition, or mummified [23]. The sexual maturity (mature or immature) was determined based on species-specific standard TL ranges and sex [24–29]. In addition, histological gonadal features, and the lactation and uterine conditions of females were evaluated and documented.

Nutritional status was estimated based on anatomical parameters (e.g. prominent bones, dorso-axial muscular mass and the amount of fat) and classified as good, moderate or emaciated. The study was performed only with stranded animals. No animal was intentionally caught or killed. Consequently, submission to a Brazilian institutional ethics committee on animal usage was not required. Nevertheless, a field permit was granted by the Ministry of Environment—MMA (SISBIO 18688–1, 2, 3).

### Gross, histological and immunohistochemical analysis

Routine necropsies were performed using standard protocols [23], except for the nervous system. Neurological analyses was not attempted, partly owing to tissue autolysis/decomposition, but also intact skull was required for ancillary morphological evaluations. Therefore, no neurological relationships between neurological status and cause of death (COD) were established.

Tissue samples (skin, muscle, lymph nodes, spleen, lung, kidney, adrenals glands, gonads, urinary bladder, heart, tongue, esophagus, stomach, intestine pancreas and liver) for histopathological analysis were fixed in 10% buffered formalin solution, routinely processed, embedded in paraffin, and stained with hematoxylin and eosin. Additionally, histochemical techniques including the Masson’s trichrome (for tissue fibrosis), periodic acid-Schiff (for glomerular basement membrane), Grocott (for fungi), Gram (for bacteria), and Ziehl-Neelsen (for acid-fast bacteria) were used to better characterize observed lesions. Immunohistochemical analysis was performed on selected lung tissue sections as described by Headley et al. [30] using a monoclonal anti-canine distemper virus (CDV) antibody (1:1000, VMRD, Pullman, Washington).

Animals were examined for parasites and where collected, specimens were fixed in 5% formalin or maintained in 10% alcohol, clarified in glycerin or amann lactophenol, and mounted onto histological slides for evaluation with an optical microscope. The species identification followed Altrão et al. [31].

### Evaluation of mortality

The COD and/or stranding was postulated based on epidemiological data, necropsy, histopathology, and immunohistochemical findings. However, in those cases where microscopic analyses were not possible, detailed gross descriptions were used to categorize COD. Cases of bycatch were diagnosed and classified according to Moore et al. [6] with some modifications. More specifically, we ascribed the following categories and criteria: (i) *confirmed*: reported by fishers or obvious cutaneous net marks of affected animals; (ii) *probable*: presence of recent rope/line marks at the skin, froth in airways and pulmonary congestion (including microscopic assessment), amputation and body slit and a good nutritional status; or (iii) *suspected*: animals with advanced decomposition and fishing net wrapped around the peduncles.

### Statistical analyses

While all findings are quantitatively presented, owing to sample size, formal statistical analyses were limited to *S*. *guianensis*. Specifically, contingency table analyses using the Fisher’s exact test (two-tailed) were used to test hypothesis that the frequencies of mortalities (between natural and all anthropogenic deaths or natural and a subset of bycatch deaths) were independent of sex and maturity (mature and immature) and nutritional status. The same analyses were used to evaluate if there was an association between lung angiomatosis and parasitic pneumonia or sexual maturity. Excel Action 2.6 and R statistical software version 2.5.1 were used for statistical analysis. The alpha level was set at 0.05.

## Results

A total of 218 stranded cetaceans from 11 identified species were recorded between 2007 and 2012 (Fig 1). The most frequent species included *S*. *guianensis* (n = 155), *T*. *truncatus* (n = 23) and *P*. *blainvillei* (n = 17). Other less common species were *S*. *frontalis* (n = 5), short-beaked common dolphin (*Delphinus delphis*, n = 3), humpback whale (*Megaptera novaeangliae*, n = 3), minke whale (*Balaenoptera acutorostrata*, n = 3), Bryde’s whale (*Balaenoptera edeni bridey*, n = 2), *Balaenoptera* spp. (n = 2), long-finned pilot whale (*Globicephala melas*, n = 2), spinner dolphin (*Stenella longirostris*, n = 1), and rough-toothed dolphin (*Steno bredanensis*, n = 1). One mummified, small odontocete remained unidentified (S1 Table).

Of the above animals, 57 (26.1%) were grossly evaluated, but owing to advanced autolysis/decomposition, only 50 animals had samples collected and were histologically evaluated. Immunohistochemical analysis was performed on selected lung tissue sections from 9 animals that presented adequate epithelium conservation. The difference between the total number of cetaceans sampled and the total number evaluated was primarily due to (i) advanced *post-mortem* autolysis or mummified carcasses (131/218), and (ii) logistics, including environmental conditions.

The 57 necropsied individuals comprised six species, including *S*. *guianensis* (n = 50), *P*. *blainvillei* (n = 2), *S*. *frontalis* (n = 2), *S*. *longirostris* (n = 1), *T*. *truncatus* (n = 1) and *G*. *melas* (n = 1). Of these carcasses, 25 (43.8%) carcasses were classified as fresh and 20 (35.1%) moderately decomposed, while 12 (21.1%) had advanced decomposition. The animals included 28 males, 27 females, and two of unknown sex, with 33 mature and 24 immature. The nutritional status was classified as good in 32, moderate in 9 and emaciated in 4 animals (12 were not assessed).

**Fig 2:**
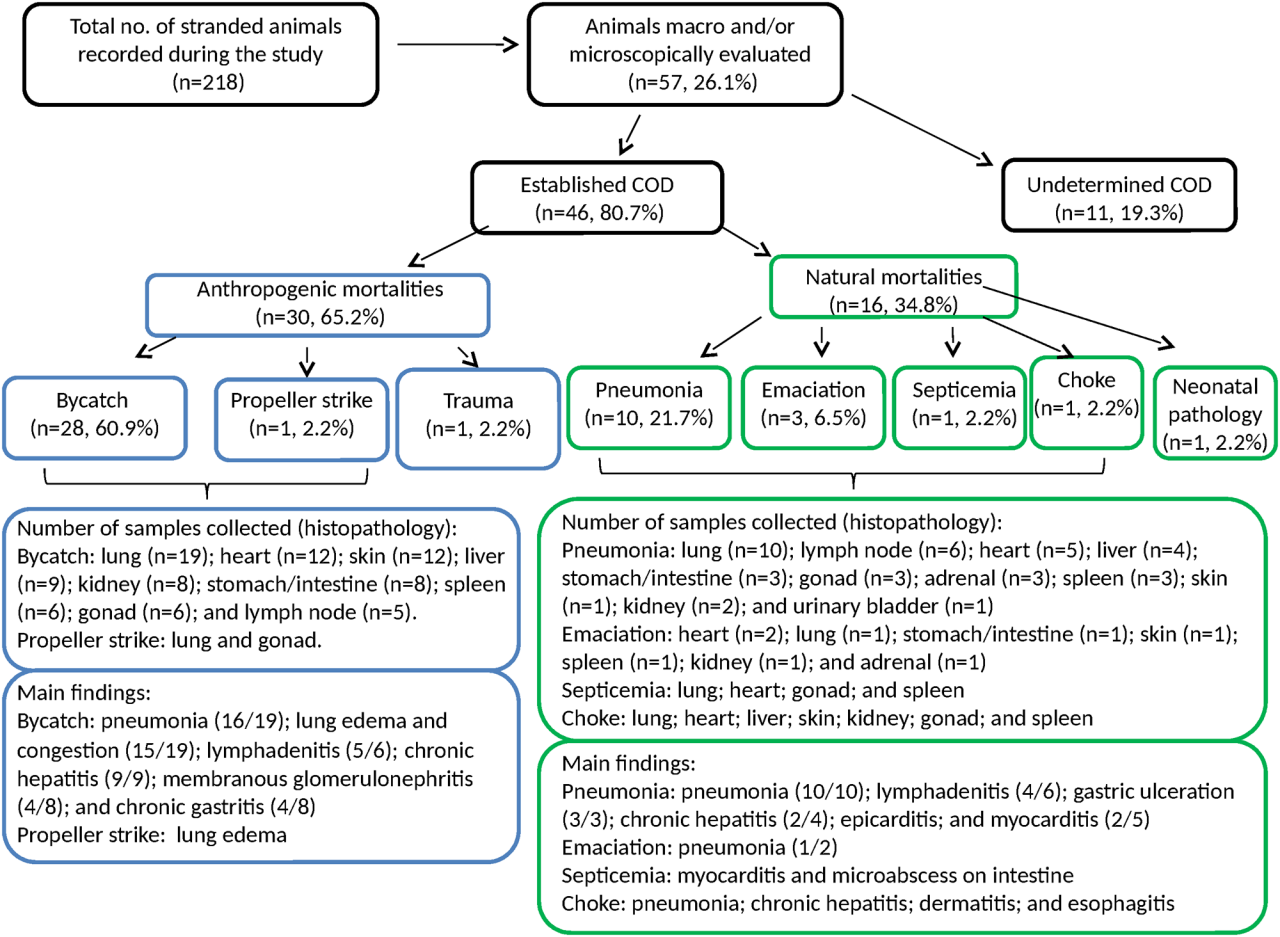
Diagram of small cetacean mortalities, with the numbers of carcasses recovered, diagnosed, and the collected samples and lesions according to different causes of death.

The COD was determined for 46 of the 57 (80.7%) necropsied animals. Anthropogenic pathological causes (30/46; 65.2%) were more frequently detected than those that were natural (16/46; 34.8%) (Fig 2). Although the COD was not determined for 11 animals (4 fresh and 7 with advanced decomposition), pathological findings and etiological agents were nevertheless identified (S2 Table). Individual life history data and pathological findings (species, sex, sexual maturity, stages of decomposition, parasitic and microbiological exam, gross and histological findings, and the COD) are listed in S2 Table.

Across all anthropogenic and natural mortalities among *S*. *guianensis*, the COD was independent of sex (natural COD: 8 females and 6 males; anthropogenic COD: 14 females and 12 males; Fisher’s exact test, p = 1.00), maturity (natural COD: 8 mature and 6 immature; anthropogenic COD: 19 mature and 8 immature; Fisher’s exact test, p = 0.49) and nutritional status (natural COD: 7 good, 5 moderate and 2 emaciated; anthropogenic COD: 19 good, 3 moderate and 1 emaciated; Fisher’s exact test, p = 0.11). Because bycatch was the most common anthropogenic COD among *S*. *guianensis* (i.e. 25/46 deaths), contingency tables were re-assessed with this reduced data set. However, similar to the full analyses, COD remained independent of sex (bycatch COD: 14 females and 10 males; natural COD: 8 females and 6 males; Fisher’s exact test, p = 1.00), maturity (bycatch COD: 18 mature and 7 immature; natural COD: 8 mature and 6 immature; Fisher’s exact test p = 0.48), and nutritional status (bycatch COD: 18 good, 3 moderate and 1 emaciated; natural COD: 7 good, 5 moderate and 2 emaciated; Fisher’s exact test, p = 0.12). Detailed information concerning natural and anthropogenic COD is provided below.

### Anthropogenic mortalities

Fishery bycatch was the most frequent COD in this category (28/46): confirmed in 24, probable in three and suspected in one of the diagnosed animals. *Sotalia guianensis* comprised 89.3% of bycatch cases. The carcasses of bycaught specimens mostly were recovered fresh, and less frequently with moderate or advanced decomposition.

**Fig 3:**
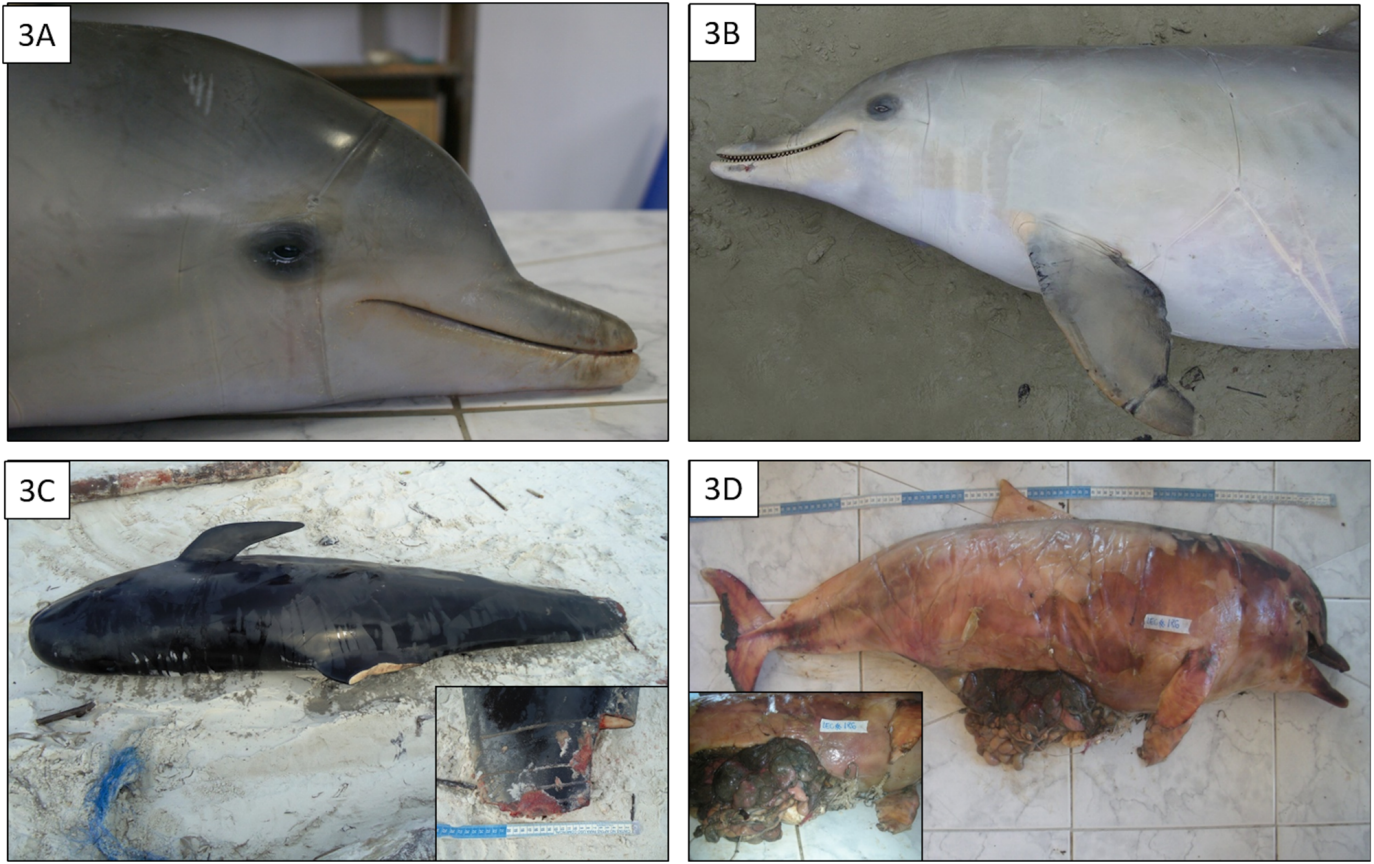
Macroscopic findings supporting evidence of interactions between *Sotalia guianensis* and anthropogenic activities. A and B: Impression from gillnet entanglement at the head, behind the eyes, fin and encircling the thoracic and abdominal regions. C: Fluke amputation and haemorrhage. D: A hook deeply embedded in the mouth, and line encircling the body to the abdomen producing multifocal hematoma, haemorrhage and trauma with evisceration and intestine perforation (insert).

The main gross lesions were superficial and deep bruises caused by contact with ropes or fishing nets encircling the body or extremities (mouth, fins, and tail), including 24 *S*. *guianensis*, 1 *P*. *blainvillei* and 1 *S*. *frontalis* (Fig 3A and 3B). Deep and linear wounds that penetrated the body cavity or were proximal to amputated fluke and fins were also observed in 6 *S*. *guianensis* and 1 *G*. *melas* (Fig 3C). The latter lesions may have been inflicted by netting and/or knives (possibly used to disentangle animals).

Other anthropogenic COD involved 2 *S*. *guianensis*. In the first case, the animal had a hook deeply embedded in its mouth and rope lines encircling its body, resulting in multifocal abdominal hematomas, haemorrhages, and trauma with evisceration and intestinal perforation (Fig 3D). In the second case, a single sharp traumatic injury that affected the skin, subcutaneous and deep muscular planes was observed, producing a severe spinal fracture at the dorsal peduncle, and was probably caused by propeller.

**Fig 4:**
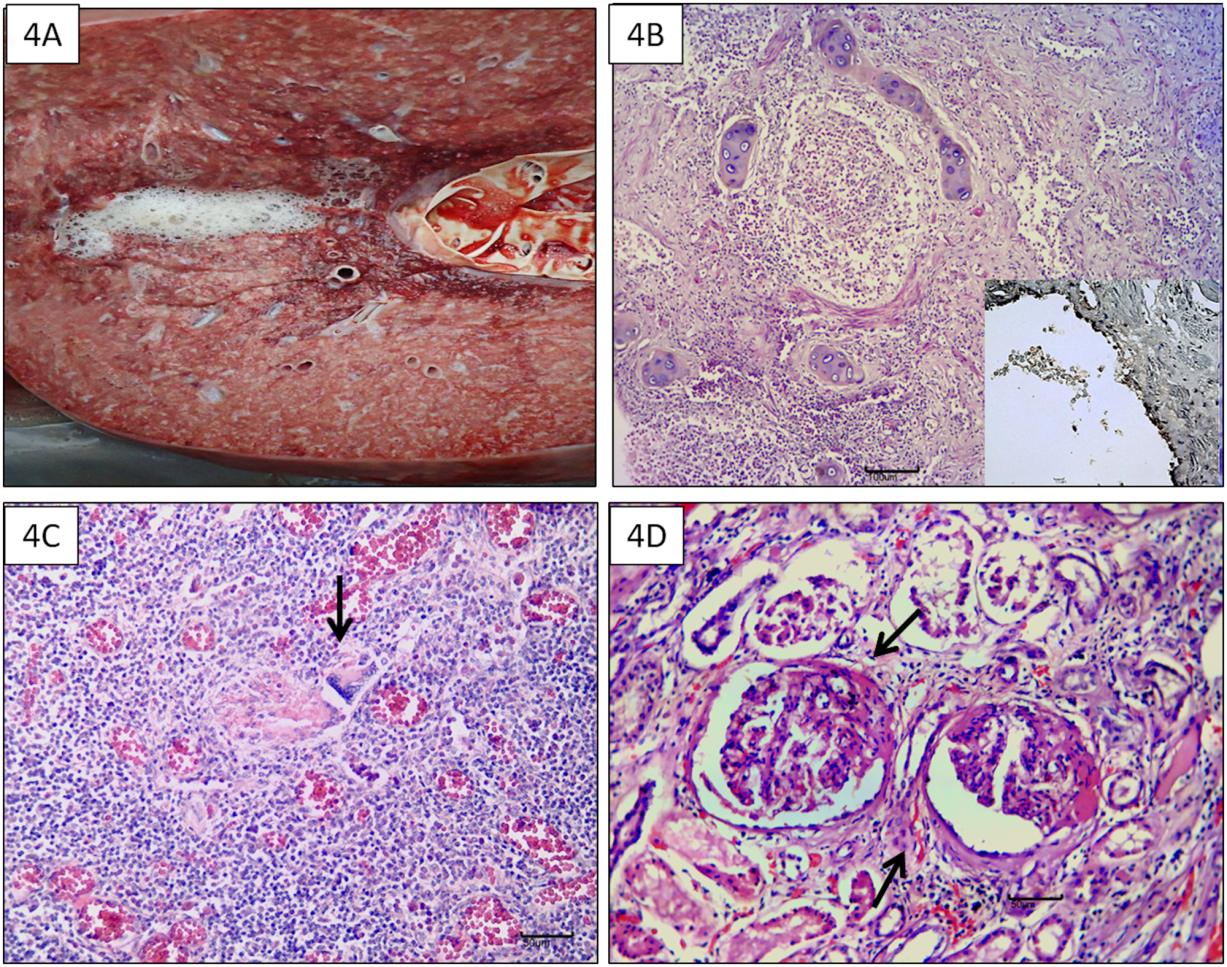
Macroscopic and microscopic findings associated with inflammatory processes. A: *Globicephala melas*: Pulmonary edema. B: *Sotalia guianensis*: Chronic bronchointerstitial pneumonia associated with morbillivirus infection and *Halocercus brasiliensis* (not in the figure) (HE, 4x) and positive immunostaining for CDV (insert, 40x). C: Granulomatous lymphadenitis with giant cells (arrow) (HE, 20x). D: Membranous glomerulonephritis (arrows) (HE, 20x).

In addition, other important findings were observed during gross and histopathological evaluation in 23 bycaught animals. These observations included severe white or clear frothy pulmonary fluid and/or mild-to-severe pulmonary edema (Fig 4A) and congestion in 17 animals. Moderate-to-severe chronic bronchointerstitial pneumonia was diagnosed in 16 *S*. *guianensis*. Pneumonia was associated with pulmonary angiomatosis in 11 animals and granulomatous pneumonia in 4.

The principal etiological agent associated with pneumonia was *H*. *brasiliensis* (10/16 animals). However, for 2 animals presenting *H*. *brasiliensis* there were concomitant morbillivirus (Fig 4B) and bacterial infections, respectively. Grossly multifocal bronchiolar occlusion by nematodes was observed in 5 cases and histopathological evaluation revealed moderate-to-severe chronic bronchointerstitial or granulomatous pneumonia and fibrosis in all cases. Mild-to-moderate *Anisakis* spp. (Nematode: Anisakidae: Ascarideoidea) and *Braunina cordiformis* (Trematoda: Brauninidae: Diplostomoidea) infestations were observed in 5 and 2 main stomachs, respectively, of 11 evaluated *S*. *guianensis*. Gastric parasitism was associated with moderate chronic gastritis and focal calcification of gastric mucosa in 4 animals.

Another frequent finding was granulomatous and caseous lymphadenitis which occurred on the mesenteric, mediastinal, and cervical lymph nodes of 5 *S*. *guianensis* with angiomatosis (Fig 4C). Lymphoid depletion was observed in 3 animals, including 1 case with morbillivirus-induced bronchointerstitial pneumonia. Histochemical techniques used to identify the presence of possible intralesional bacteria, mycobacteria and/or fungi were negative.

Additional histopathological findings in *S*. *guianensis* included membranous glomerulonephritis (4 cases; Fig 4D) and chronic epicarditis (3 cases) were observed. Chronic dermatitis was observed in 3 *S*. *guianensis* and a *G*. *melas* comprising multifocal dark-fringed spots (pale in the center and with darker edges) at the submandibular skin, fluke, and fins; multifocal ulcerated areas on the lateral peduncle; or black (small, rounded and, slightly depressed) and multifocal irregular lesions (whitish, well circumscribed, with a velvety appearance) across the pectoral and abdominal lateral regions.

### Natural mortalities

**Fig 5:**
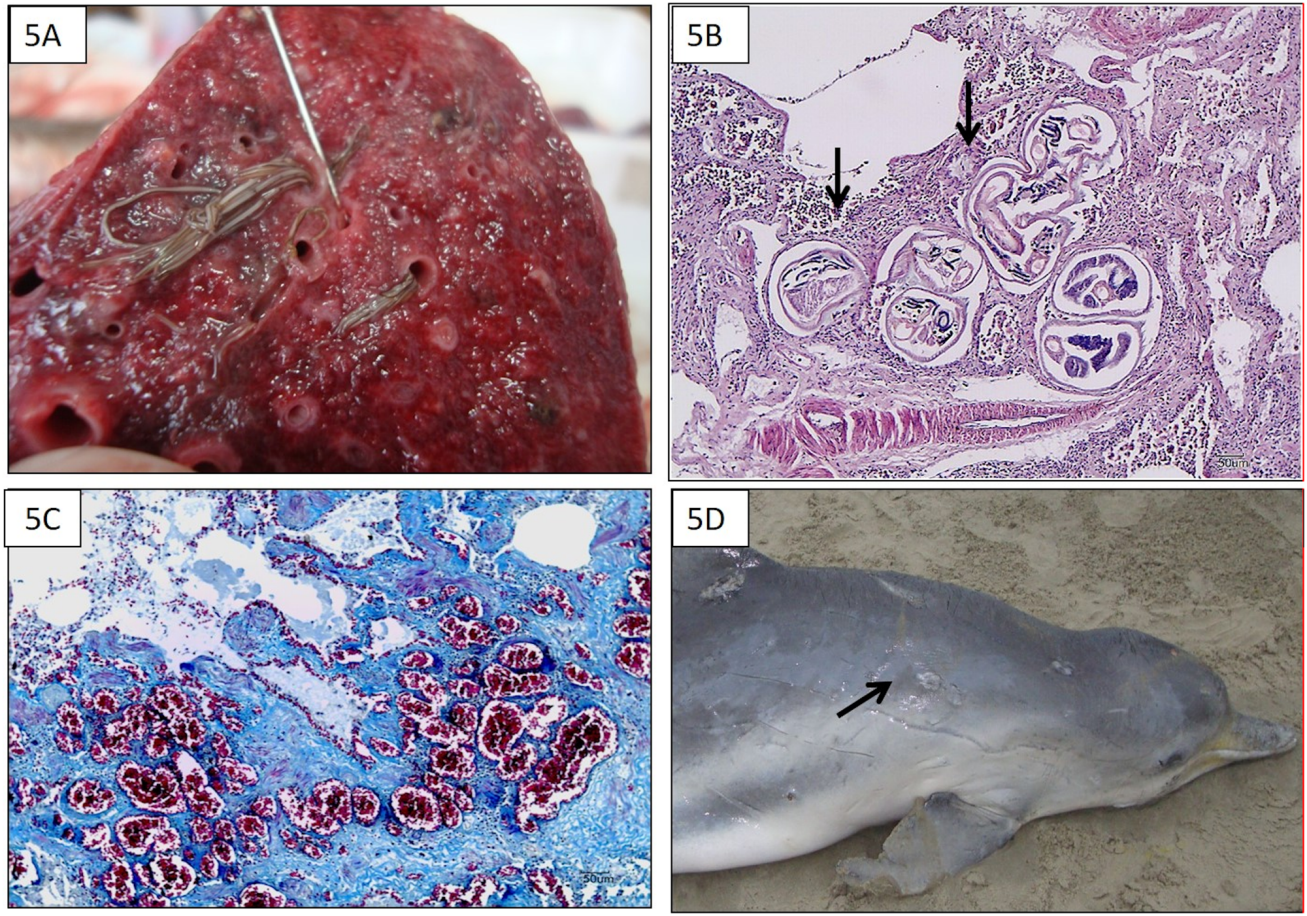
Macroscopic and microscopic findings of *Sotalia guianensis* that died of natural causes. A: *Halocercus brasiliensis* in bronchi and bronchiole lumens, with pneumonia the cause of death (COD). B: Chronic bronchointerstitial pneumonia caused by adults *H*. *brasiliensis* (arrows) (HE, 10x). C: Lung angiomatosis (Masson’s trichrome, 20x). D: Emaciation COD, muscular atrophy of dorsal cephalic musculature, multifocal ulcerated areas on skin (arrows).

Of the 46 animals with their COD determined, 16 were classified as incurring natural mortalities. Specifically, pneumonia was the established COD in 9 *S*. *guianensis* and 1 *S*. *longirostris*, with associated parasitic infestation by *Halocercus brasiliensis* (Nematoda: Pseudaliidae: Metastrongyloidea) in 7 *S*. *guianensis* (Fig 5A); 2 of these animals were concomitantly infected by a morbillivirus. One *S*. *guianensis* had pneumonia caused by cocci Gram negative bacteria. Severe parasitic pneumonia was observed in 2 cases, with multifocal bronchiolar occlusion by adults, severe fibrin deposition on the pleura, severe focal emphysema, and multifocal parasitic granulomas. Histopathology revealed necrotizing parasitic granulomatous pneumonia with congestion, edema, and hemorrhage associated with nematode presence. Larvae and adult nematodes were identified within the bronchi, bronchiole (Fig 5B) and alveoli. Occasionally, granulomas containing cores of degenerate lungworms were also noted. Mild-to-moderate eosinophils, macrophages and lymphocytes infiltrations were observed to thicken the interstitium. Occasionally, edema filled the bronchioles and alveoli lumen.

Angiomatosis was another frequent pulmonary lesion (9/10 animals), characterized by different-sized, thick-walled blood vessels within the visceral pleura and interstitium (Fig 5C). The condition occurred independent of sexual maturity (Fisher’s exact test, p = 0.62). In severe cases, the clusters of newly formed small caliber vessels became (i) increasingly collagenized, producing vessels that formed small collagenous islands or muscular islands in a fibrotic nodule, and/or (ii) formed a thin cordlike pattern resulting in ruptured vessels and confluent areas. The cause of these vascular lesions was evaluated despite COD, and was significantly associated with the presence of parasites (Fisher’s exact test, p = 0.03), and independent of sexual maturity (Fisher’s exact test, p = 0.62). Other histopathological findings accompanying pneumonia as COD are given in S2 Table.

Emaciation was determined as the COD for 2 *S*. *guianensis* and 1 *P*. *blainvillei* (3/46); 1 animal of each species was a dependent calf. Emaciation was characterized by muscular and adipose tissue atrophy, with consequent postcranial depression and clearly defined rib impressions under the skin (Fig 5D), and empty stomachs. Chronic bronchointerstitial pneumonia and *H*. *brasiliensis* infestation were observed in 1 animal. Neonatal disease was diagnosed in one *S*. *guianensis* calf (1/46). All of these animals had multifocal ulcerative and granulomatous dermatitis mostly confined to the edges with variable purulent exudate and hemorrhage. In the neonatal related COD, according to a fisher who had recovered the carcass, the cutaneous lesions were caused by the mother, or other group members, trying to maintain the animal in motion or to make it surface, following an epimeletic behavior. Based on gross examination, findings were restricted to a hemorrhagic intestinal ulcer and hemorrhagic spleen.

Septicemia was determined in 1 *S*. *guianensis* in good nutritional status (1/46). Histopathology revealed multifocal bacterial emboli, severe pneumocyte necrosis, diffuse inflammatory infiltrate throughout lung tissue and co-infection by *H*. *brasiliensis*. This animal also had focal suppurative myocarditis and multifocal microabscesses in their small intestine. The remaining natural COD included choking in 1 *S*. *guianensis* in moderate nutritional status (1/46). Specifically, a 77- cm long teleost was observed obstructing the lumens of the esophagus and larynx. Other findings included multifocal cutaneous erosions and irregular whitish well circumscribed areas on the head, abdomen, and fluke dorsal area.

Because nervous systems were not evaluated, any associated pathology cannot be eliminated as contributing towards the observed mortalities, especially in those cases of morbillivirus detection (1 bycaught and 2 pneumonia COD). Notwithstanding this limitation, the key pathological observations among natural and anthropogenic mortalities were considered sufficiently severe to evoke death.

## Discussion

Assessing marine mammal health is challenging, but the utility in terms of insight into marine-ecosystem quality is evidenced here through delineation of natural and anthropogenic (direct and indirect) pathological categories [2,32,33]. The observations can be discussed by first considering the likely ultimate COD, followed by other concomitant, important pathological findings, and then used to propose conservation actions.

Bycatch was the main COD among stranded cetaceans in this study (~61%). Similar results have been recorded during previous studies conducted off northeastern (~52%) and southeastern Brazil (76%) [34,35], reiterating bycatch as a threat to cetacean populations worldwide [5]. One of the key fishing gears used in southern Brazil is gillnets [36,37], which in many areas pose a conservation threat to several populations of marine mammals [38,39], especially *S*. *guianensis* and *P*. *blainvillei*; both of which historically are the most common, regionally caught cetaceans [40,41]. According to the results of the present study and interviews with fishers, *S*. *guianensis* and *P*. *blainvillei* maintain a strong vulnerability off Paraná [42,43]. The associated mortalities are of concern because they might negatively impact on reproduction potential and ultimately the species survival [5].

Many of the evaluated carcasses were freshly decomposed, facilitating concise pathological evaluations. A correlation between the abundance of *S*. *guianensis* and high fishing pressures was apparent and provided subsequent insight into pre-mortality trauma; which was especially relevant for *S*. *guianensis* (predominantly stranded as a consequence of fishery bycatch).

Necropsy and histopathological examinations are valuable tools for diagnosing diseases and COD [32]. In this study, gross findings on the skin (e.g. net marks, peri-mandibular bruises, amputations, body incision, and hemorrhages) and lungs (e.g. persistent froth and congestion) were observed in most bycaught animals and were considered significant indicators of entanglement [6]. However, it is important to note that obvious net marks are not required to conclude a diagnosis of fishery bycatch, since trawl-caught animals might be protected from mesh contact by other catches in the codend [6,44].

Vessel collisions are another anthropogenic impact that have adverse effects on cetaceans, and typically are observed as sharp traumatic injuries and blunt force trauma [6]. Sharp traumatic injuries were herein observed in one *S*. *guianensis* and previously have been recorded among other small cetaceans and whales in the Southern Hemisphere [45]. Such injuries can have direct (death) or indirect (non-fatal lesions, but nevertheless associated with pathological agents and secondary diseases) consequences for cetacean health [6,7], and might be more prevalent in harbors (such as the PEC) and adjacent areas, owing to high traffic of recreational, artisanal and commercial fishing vessels.

Notwithstanding the attribution of many fatalities due to anthropogenic causes, the gross and histopathological evaluation of internal organs also clearly demonstrated several animals had coexisting or underlying disease processes of natural origins and with variable severity. The clinicopathological relevance of such findings remains uncertain. However, conceivably individuals with severely affected vital organs (e.g. the lungs), could develop chronic, debilitating diseases and/or become predisposed to fishing interactions as a consequence of a diminished ability to avoid and disentangle from gear, or increased susceptibility of entanglement during depredation.

Of the pre-existing conditions, chronic bronchointerstitial pneumonia was the most frequent lesion among animals diagnosed with both anthropogenic and natural COD, and was frequently associated with partial obstruction by *H*. *brasiliensis* in pulmonary airways. This parasite affects the lower respiratory tract of several cetaceans [32,46,47] and associated infestations have been related to severe manifestations of dyspnea, moderate-to-severe pneumonia and death in *S*. *guianensis* and other cetaceans throughout Brazil [34,48,49]. Pulmonary nematode infestations were characterized by lesions that varied from interstitial pneumonia (in larval migration) to chronic bronchitis, caused by intrabronchial and/or intrabronchiolar adult lung worms [50].

Symptoms of lungworm infestation and its association with stranding and mortality vary among host and parasite species, as well as the intensity of infection [47,51]. For example, Fauquier et al. [46] observed that lesions associated with *Halocercus lagenorhynchi* and *Skrjabinalius cryptocephalus* were not sufficiently severe to be of clinical importance in *T*. *truncatus* off Florida, but only 4 animals had their parasite numbers evaluated (9–59 lungworms). In another study, debilitating conditions affecting the respiratory capacity and diving ability were observed in cetaceans off northeastern Brazil (2–122 lungworms) [47].

Although the intensity of parasitism by *H*. *brasiliensis* was not evaluated here, a previous report describing bycaught *S*. *guianensis* presenting pneumonia suggested the number of parasites in bronchiolar lumen varied greatly—from 3 to more than 200 nematodes [31]. Considering the above, efforts to assess parasitic burdens, or intrinsic features of parasitic and host interactions, should be improved so the severity of pathological findings and the consequent health impacts of *H*. *brasiliensis* on *S*. *guianensis* and other cetacean species can be better understood.

A frequent concomitant alteration observed on parasitized lungs of *S*. *guianensis* is angiomatosis. In the present study, a strong association between pulmonary angiomatosis and *H*. *brasiliensis* was noted. Similarly, this alteration has also been linked to pulmonary nematodiasis in other cetacean [52,53]. It is also known that cetaceans have a rich vascular network within their respiratory system [54] and, like for humans, a natural increase in vascularization (e.g. angiogenesis/vasculogenesis) relative to ontogenetic process (from birth to adult life) could be hypothesized [55]. However, no positive association was observed between vascularization and sexual maturity here. These results suggest pulmonary infestation by *H*. *brasiliensis* induces tissue injury and angiomatosis is part of the host response. The mechanisms involved in parasite-host tissue response remain cryptic [52].

Morbillivirus infection affects cetacean populations and was first diagnosed in a *S*. *guianensis* in Brazil in 2010 [17]. Here we observed bronchointerstitial pneumonia associated with morbillivirus and *H*. *brasiliensis* co-infection in 3 animals. These results may indicate the virus has been circulating among *S*. *guianensis* along the Brazilian coastline over the last few years. We currently are evaluating the molecular and epidemiological findings associated with morbillivirus of *S*. *guianensis* off the Paraná coastline to determine the potential impacts of this emerging disease. Morbilliviruses could be a key threat to *S*. *guianensis* populations and so understanding the associated distributions is a prerequisite for assessing habitat degradation and to drive conservation actions [9, 40].

Beyond systemic virus infections, lymphoid depletion has been related to chronic infections, starvation and the immunotoxic effects of chronic exposure to environmental contaminants [18,56]. We observed chronic infections in *S*. *guianensis* with lymphoid depletion, especially granulomatous lymphadenitis, which was frequently noted in peribronchial lymph nodes. The associated histological features were similar to those reported for bacterial and parasitic infection in harbor porpoises (*Phocoena phocoena*) stranded in northern Europe [57,58] and also observed here in 1 animal with a morbilliviral infection.

Another potential cause of lymphoid depletion is chronic toxic exposure. Specifically, a noteworthy observation here was membranous glomerulonephritis and glomerular atrophy. The same lesions were previously observed in 22% of cetaceans collected during an earlier Brazilian study [59] and have been associated with elevated levels of cadmium, copper, and zinc in cetaceans [60,61]. Nevertheless, toxicological analyses were not performed here, thus the levels of trace elements and their possible associations with lymphoid depletion and chronic renal changes remain speculative.

The results of the pathological examinations of bycaught animals revealed severe lesions (bronchointerstitial pneumonia, lymphadenitis and glomerulonephritis) of natural origins. This reiterates the value of necropsies and histopathological analysis, particularly among bycaught animals, which might be expected to be healthy given their good nutritional status and evidence of active feeding (e.g. non or partially digested food within the first gastric (keratinized) chamber). A larger sample size would facilitate a better understanding of the epidemiological aspects of bycaught animals, including any interactions between underlying health and the ability to withstand fishery interactions [6,32,45,62].

This study clearly increases the body of knowledge concerning baseline pathology and COD among cetaceans in southern Brazil. However, it is important to consider that while strandings can improve our understanding of occurrence frequencies, key demographic parameters, and the gravity of interactions with human activities [6,33,63], the relevance of such animals to population and ecological parameters remains poorly understood. Key reasons for such knowledge gaps include unknown animal geographic origins and opportunistic sampling which can preclude robust statistical credibility [64].

Notwithstanding the limitations above, the specimens in this study mostly represented species known to be PEC residents and with established coastal and estuarine distributions (*S*. *guianensis* and *P*. *blainvillei*) [65, 66]. Further, those specimens from more diverse geographical distributions (e.g. *T*. *truncatus*, *Stenella* sp. and *G*. *melas*) which had their COD diagnosed were all freshly decomposed, suggesting their capture and death occurred close to the shoreline (although drift rates were unknown and may have affected debilitated animals before dying). Therefore, it is likely that the stranded cetaceans evaluated during this study reflected local human threats and health parameters off Paraná, reiterating the deleterious conditions faced by small cetaceans frequenting coastal urban areas worldwide.

## Conclusions

While the results also provide evidence of severe *H*. *brasiliensis*-associated pneumonia among resident cetaceans, anthropogenic impacts were identified as the leading COD among cetaceans stranded along the Paraná coast. Consequently, future conservation policies should be based on improved monitoring of fishing activities (considering the mortality of ~3 animals month^–1^ across one estuary and 100 km of coastline), including a review of the current legislation and perhaps focused efforts towards developing bycatch mitigation strategies and intense fishing-community education programs. Quantifying other indirect threats, including chemical contamination, habitat degradation and underwater noise is also necessary, because these impacts can evoke the types of stress, immunosuppression and diseases observed during our investigation.

Although the studied area is considered a biosphere reserve by UNESCO and a biodiversity hotspot, the results presented here indicate local cetaceans are susceptible to hazards associated with anthropogenic activities and severe diseases potentially arising from local disturbances. Cetaceans are sentinels of marine ecosystems. Consequently, ongoing monitoring and efforts at reducing anthropogenic impacts among these species could be important tools towards a broader environmental conservation at this World Heritage site.

## Supporting Information

S1 Table Species identification, decomposition stage, samples for gross analysis and mortality causes among cetaceans stranded on the Paraná coast, southern Brazil. 2: fresh, 3: moderate decomposition, 4: advanced decomposition, 5: mummified, NI: not informed, COD: cause of death.
(DOCX)

S2 Table Individual information concerning cetaceans stranded on the Paraná coast, southern Brazil. F: female; M: male; SM: maturity stage; I: immature; M: mature; 2: fresh, 3: moderate decomposition, 4: advanced decomposition; NE: not examined; ^1^ parasites identified by Altrão et al. [33]; *Confirmed by histological evaluation.
(XLSX)
