# Supplementary material for: Assessing Disease and Mortality among Small Cetaceans Stranded at a World Heritage Site in Southern Brazil
Source: PLoS One. 2016 Feb 12;11(2):e0149295. doi: 10.1371/journal.pone.0149295 (PMC4752507; doi:10.1371/journal.pone.0149295)
Supplement: S1 Table — 2: fresh, 3: moderate decomposition, 4: advanced decomposition, 5: mummified, NI: not informed, COD: cause of death. (DOCX) [file pone.0149295.s001.docx]

|  | Decomposition stage | |  |  |  |
| --- | --- | --- | --- | --- | --- |
| Species | **2 and 3** | **4 and 5** | **Total Sample** | **Gross analysis** | **Established**  **COD** |
| *Balaenoptera acutorostrata* | 1 | 2 | 3 | 0 | 0 |
| *Balaenoptera* sp. | 0 | 2 | 2 | 0 | 0 |
| *Delphinus delphis* | 0 | 3 | 3 | 0 | 0 |
| *Globicephala melas* | 1 | 1 | 2 | 1 | 1 |
| *Megaptera novaeangliae* | 1 | 2 | 3 | 0 | 0 |
| *Pontoporia blainvillei* | 5 | 12 | 17 | 2 | 2 |
| *Sotalia guianensis* | 70 | 85 | 155 | 50 | 40 |
| *Stenella frontalis* | 2 | 3 | 5 | 2 | 1 |
| *Stenella longirostris* | 1 | 0 | 1 | 1 | 1 |
| *Steno bredanensis* | 0 | 1 | 1 | 0 | 0 |
| *Tursiops truncatus* | 5 | 18 | 23 | 1 | 1 |
| *Balaenoptera edeni bridey* | 0 | 2 | 2 | 0 | 0 |
| NI | 0 | 1 | 1 | 0 | 0 |
| Total | **86** | **132** | **218** | **57** | **46** |
